# Efficacy and Safety of Enlicitide in Hypercholesterolemia: A Systematic Review and Meta‐Analysis of Randomized Controlled Trials

**DOI:** 10.1002/edm2.70358

**Published:** 2026-10-13

**Authors:** Muhammad Farhan, Muhammad Murtaza, Muhammad Mujtaba Rasool, Farah Khan, Hammad Amjad, Kuldeep Dalpat Rai, Sheikh Muhammad Ebtehaj Ali, Ahmed Asad Raza, Muhammad Ahmad Sohail, Abedin Samadi, Furqan Hassan

**Affiliations:** ^1^ Karachi Medical and Dental College Karachi Pakistan; ^2^ Services Institute of Medical Sciences Lahore Pakistan; ^3^ Allama Iqbal Medical College Lahore Pakistan; ^4^ Dow University of Health Sciences Karachi Pakistan; ^5^ Department of Medicine Abbasi Shaheed Hospital Karachi Pakistan; ^6^ Jinnah Sindh Medical University Karachi Pakistan; ^7^ Shifa College of Medicine Islamabad Pakistan; ^8^ Kabul University of Medical Sciences Abu Ali Sina Kabul Afghanistan; ^9^ Nishtar Medical University Multan Pakistan

**Keywords:** enlicitide, hypercholesterolemia, LDL cholesterol, meta‐analysis, MK‐0616, PCSK9 inhibitor

## Abstract

**Background:**

Atherosclerotic cardiovascular disease remains a leading cause of global mortality, with low‐density lipoprotein cholesterol (LDL‐C) reduction being the cornerstone of prevention. Despite effective therapies, many high‐risk patients do not achieve recommended LDL‐C targets. Enlicitide (MK‐0616), an oral proprotein convertase subtilisin/kexin type 9 (PCSK9) inhibitor, offers a potential alternative to injectable PCSK9‐directed therapies.

**Objective:**

To evaluate the efficacy and safety of enlicitide in adults with hypercholesterolemia and assess its dose‐dependent effects on lipid parameters.

**Methods:**

We conducted a systematic review and meta‐analysis of randomized controlled trials (RCTs) according to PRISMA 2020 guidelines. PubMed, Embase, Scopus, ClinicalTrials.gov, and Google Scholar were searched from inception through March 2026. Random‐effects meta‐analysis using restricted maximum likelihood estimation was performed. The primary outcome was the percentage change in LDL‐C from baseline. Secondary outcomes included non‐high‐density lipoprotein cholesterol (non‐HDL‐C), apolipoprotein B (ApoB), lipoprotein(a), triglycerides, and safety outcomes. Risk of bias and certainty of evidence were assessed using RoB 2.0 and GRADE.

**Results:**

Three placebo‐controlled RCTs involving 3593 participants were included. Enlicitide significantly reduced LDL‐C compared with placebo (mean difference [MD], −56.19%; 95% CI, −64.14 to −48.25; *p* < 0.0001), with moderate heterogeneity (*I* ^2^ = 56.9%). Significant reductions were also observed in non‐HDL‐C (MD, −51.36%), ApoB (MD, −47.09%), lipoprotein(a) (MD, −20.10%), and triglycerides (MD, −13.12%). A numerical dose–response gradient was observed, although subgroup differences were not statistically significant. Sensitivity analyses supported the robustness of the findings. Rates of adverse events, serious adverse events, and treatment discontinuation were comparable between groups.

**Conclusion:**

Enlicitide produced substantial reductions in LDL‐C and other atherogenic lipid parameters without an apparent increase in adverse events. However, findings should be interpreted cautiously given the limited number of trials, moderate heterogeneity, and reliance on surrogate endpoints. Dose–response, duration‐related lipoprotein(a), and triglyceride findings were exploratory and substantially driven by single trials. Long‐term cardiovascular outcomes trials are needed to establish clinical benefit.

## Graphical abstract

Enlicitide (MK‐0616), an oral macrocyclic peptidyl PCSK9 inhibitor, blocks the interaction between circulating PCSK9 and hepatic LDL receptors, enhancing LDL‐C clearance from the bloodstream. This systematic review and meta‐analysis of three randomized, double‐blind, placebo‐controlled trials (*n* = 3593) evaluated the efficacy and safety of enlicitide in patients with hypercholesterolemia. Enlicitide produced a significant reduction in LDL‐C compared with placebo (MD −56.19%; 95% CI −64.14 to −48.25; *p* < 0.0001), with parallel reductions in non‐HDL‐C (−51.36%), apolipoprotein B (−47.09%), lipoprotein(a) (−20.10%), and triglycerides (−13.12%). A numerical dose–response gradient was observed across doses (6–30 mg), though subgroup differences were not statistically significant. Safety outcomes including adverse events, serious adverse events, and treatment discontinuation were comparable between enlicitide and placebo over 24–52 weeks. These findings support enlicitide as a clinically promising oral therapeutic option for LDL‐C lowering, with long‐term cardiovascular outcomes data pending from the ongoing CORALreef Outcomes trial.

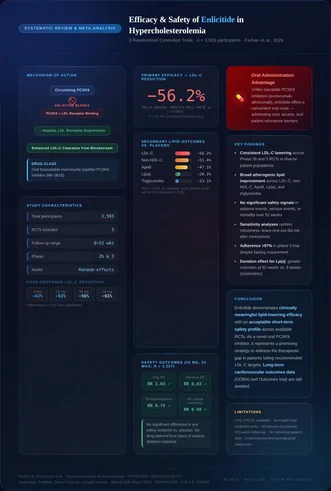

**Abbreviations**
ApoB: apolipoprotein B
ASCVD: atherosclerotic cardiovascular disease
CI: confidence Interval
GRADE: Grading of Recommendations Assessment, Development and Evaluation
IV: inverse variance
LDL‐C: low‐density lipoprotein cholesterol
Lp(a): lipoprotein (a)
MD: mean difference
Non‐HDL‐C: non‐high density lipoprotein cholesterol
PRISMA: Preferred Reporting Items for Systematic Reviews and Meta‐Analyses
PROSPERO: International Prospective Register of Systematic Reviews
RCT: randomized controlled trial
REML: restricted maximum likelihood
RR: risk ratio

## 1 Introduction

Atherosclerotic cardiovascular disease (ASCVD) is specified as the foremost contributor of global mortality; with a significant role of low‐density lipoprotein cholesterol (LDL‐C) levels in initiation and progression of cardiometabolic disorders, specially atherosclerosis [1, 2]. Consistently reduced levels of LDL‐C are associated with a decreased incidence of cardiovascular events, particularly in patients with established atherosclerotic cardiovascular disease and those at high‐to‐very‐high cardiovascular risk, as demonstrated in landmark statin and PCSK9‐inhibitor cardiovascular outcomes trials (e.g., FOURIER for evolocumab and ODYSSEY OUTCOMES for alirocumab) [3, 4, 5], further highlighting the clinical utility of the biomarker in disease control [3]. Significantly uprising trend of patients with high or very high cardiovascular risk continues to have LDL‐C levels above the recommended targets of international guidelines, despite of treatment advances [6].

Statins have been the foundation of lipid‐lowering therapies for a long time, while additional agents—including ezetimibe, bempedoic acid, inclisiran, and monoclonal antibody‐based PCSK9 inhibitors—played a significant role in achieving recommended LDL‐C targets [7]. Among these options, PCSK9‐directed therapies have shown that greater LDL‐C reduction is feasible and clinically meaningful, but broader uptake of injectable agents has been limited by cost, access, and the burden of subcutaneous administration [3, 6, 7]. For this reason, oral PCSK9 inhibition has emerged as an attractive strategy that may combine potent LDL‐C lowering with greater convenience and wider acceptability in clinical practice [6, 7, 8].

Enlicitide (MK‐0616) is an orally bioavailable macrocyclic peptide that binds circulating PCSK9 and blocks its interaction with the hepatic LDL receptor. By preventing PCSK9‐mediated receptor degradation, enlicitide increases LDL‐receptor recycling to the hepatocyte surface, thereby enhancing clearance of LDL‐C from the circulation [6, 7, 8, 9]. Early phase and Phase‐3 clinical trials have shown that enlicitide produces significantly reduced levels of LDL‐C and atherogenic lipoproteins, with a favourable short‐term safety profile in patients with hypercholesterolemia, including familial forms of the underlying disease [10, 11, 12].

The available enlicitide evidence is encouraging, but it remains distributed across Phase 2b and Phase 3 trials that differ in dose structure, patient population, and follow‐up duration [10, 11, 12]. Although class‐level analyses of oral PCSK9 inhibitors [6, 8] and individual trials of other agents in this class [13] suggest robust lipid‐lowering efficacy without a clear excess of adverse events, those data are broader than the individual enlicitide program and do not fully define the overall efficacy and safety profile of enlicitide itself. A recent class‐level network meta‐analysis of oral PCSK9 inhibitors [14] similarly provided pooled evidence at the drug‐class level but was not enlicitide‐specific and did not incorporate the most recent Phase 3 datasets. Importantly, the current evidence base remains centred on surrogate lipid endpoints rather than hard cardiovascular outcomes, while the dedicated cardiovascular outcomes trial for enlicitide is still ongoing [15]. Individual trials were also not designed to precisely characterize dose–response relationships or to determine whether the cumulative evidence is sufficient for reliable conclusions across all endpoints.

In the following context of study, a combined analysis of all known randomized controlled trials was conducted to evaluate the efficacy and safety of enlicitide in patients with hypercholesterolemia. Additionally, this meta‐analysis proves to be the first study to formally provide the model dose–response relationship across the evaluated dose range of enlicitide. However, the substantial proportion of individuals with high cardiovascular risk continues to fall short of recommended LDL‐C targets despite readily available lipid lowering therapies, including the underutilization of injectable PCSK9 inhibitors due to cost barriers and limited accessibility of injectable interventions. In order to address the underlying treatment gap, this systematic review and meta‐analysis is conducted to determine the clinical utility of the intervention on patients with hypercholesterolemia.

## 2 Materials and Methods

This systematic review and meta‐analysis included three randomized controlled trials (RCTs) for the evaluation of the safety profile and clinical utility of enlicitide in patients with hypercholesterolemia. Registration of protocol with the International Prospective Register of Systematic Reviews (PROSPERO) with the registration number of CRD420261357317 was conducted according to the internationally standardized criteria of PRISMA (Preferred Reporting Items for Systematic Reviews and Meta‐Analyses) 2020 guidelines [16, 17]. The complete PRISMA checklists are provided in Tables S1 and S2.

### 2.1 Database and Literature Search Strategy

A comprehensive literature search was conducted across PubMed, Embase, Scopus, and ClinicalTrials.gov as primary databases from inception through March 2026, with Google Scholar used solely for Supporting Information citation‐chasing and grey‐literature identification. The keywords used for searching were: ‘Enlicitide’, ‘MK‐0616’, ‘Hypercholesterolemia’, ‘LDL‐C’, ‘PCSK9 inhibitors’, ‘Cardiovascular risk’. The search results were obtained without any limitations or filters. No restrictions regarding language, publication status, or study design filters were applied. The complete search strategy of the study design is provided in Table S3.

### 2.2 Eligibility Criteria

#### 2.2.1 Inclusion Criteria

Eligibility of the studies was considered according to the following criteria of the study design. (1) Adults (≥ 18 years) diagnosed with hypercholesterolemia, including primary hypercholesterolemia or familial hypercholesterolemia, (2) enrolled patients with elevated LDL‐C levels despite lifestyle measures or standard lipid‐lowering therapy, (3) Both male and female participants of any ethnicity or geographic location, (4) randomized controlled trials evaluating enlicitide, either as monotherapy or in combination with background therapy, compared with placebo.

#### 2.2.2 Exclusion Criteria

Studies were excluded if they met any of the following conditions:

(1) Participant‐level exclusions generally included pregnancy or breastfeeding, active liver disease, uncontrolled hypertension or diabetes, marked hypertriglyceridemia, and other major conditions likely to interfere with safety evaluation or interpretation of lipid outcomes. (2) In HeFH‐specific settings, participants with homozygous, compound heterozygous, or double heterozygous familial hypercholesterolemia were excluded. (3) Studies not reporting relevant lipid outcomes or safety data for Enlicitide, (4) studies involving patients with homozygous or complex forms of familial hypercholesterolemia in heterozygous‐specific analyses.

### 2.3 Screening Procedure

Screening was performed by two reviewers. Reviewers independently screened the articles. After removing duplicates, screening was carried out according to the eligibility criteria for abstracts and titles, and later, full texts of the included studies. Any discrepancies during the analysis were resolved with the consultation of a third reviewer. A PRISMA flowchart of the study selection process is available in Figure 1.

**FIGURE 1:**
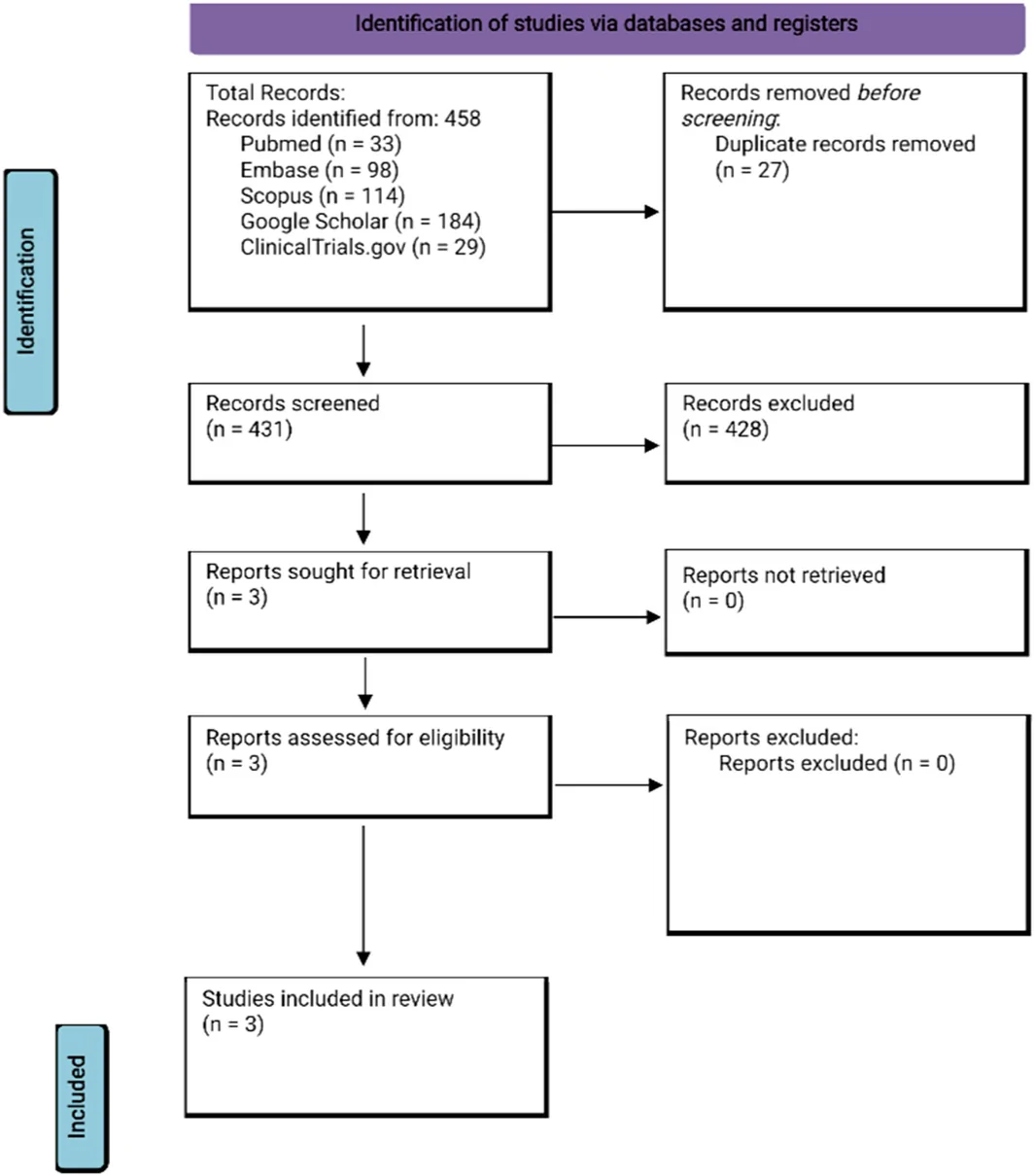
Prisma flow chart.

### 2.4 Data Extraction

Two reviewers independently performed the certainty of evidence using GRADEpro GDT software [18]. Extracted information included study characteristics (publication year, study design, country, duration, and sample size) and baseline patient data (age, sex distribution, body mass index, ethnicity, and history of ASCVD).

### 2.5 Outcomes

The percentage change in LDL‐C levels of patients with hypercholesterolemia from baseline was considered the primary objective of the study. Secondary utility of intervention is assessed with evaluation of percentage changes in non‐HDL cholesterol, apolipoprotein B, lipoprotein(a), and triglycerides. Safety endpoints of the enlicitide include the incidence of any adverse event, serious adverse events, moderate‐to‐severe adverse events, all‐cause mortality, and treatment discontinuation due to adverse event. For every efficacy outcome, we extracted the mean percentage change from baseline within each treatment arm together with its dispersion. Pooled estimates therefore represent the raw mean percentage change from baseline.

### 2.6 Risk of Bias Assessment

Two independent reviewers assessed the risk of bias of the manuscript, using Cochrane Risk of Bias 2.0 (RoB 2.0) [19]. Assessment of the manuscript covers the key domains of the study including randomization procedures, deviations from intended interventions, completeness of outcome data, outcome measurement, and selective reporting. The bias in any domain was rated as ‘low risk of bias’, ‘some concerns’ or ‘high risk of bias’, and the assessment was performed at the outcome level where applicable.

### 2.7 Certainty Assessment

Two independent reviewers performed a certain assessment of the study design to evaluate the certainty of evidence from our meta‐analysis. Five domains of the analysis including risk of bias, inconsistency, indirectness, imprecision, and publication bias were independently evaluated using GRADEpro (Grading of Recommendations Assessment, Development and Evaluation) GDT software [18].

### 2.8 Statistical Analysis

A random‐effects model was employed for all analyses. The between‐study variance (τ^2^) was estimated using the restricted maximum‐likelihood (REML) method [20]. The inverse‐variance method was used for continuous outcomes and the Mantel–Haenszel method for binary outcomes [21]. The Hartung‐Knapp adjustment was applied to random‐effects confidence intervals [22, 23]. Statistical heterogeneity was assessed using Cochran's *Q* test, the *I* ^2^ statistic, and *τ* ^2^ values [24], with *I* ^2^ thresholds of 25%, 50%, and 75% representing low, moderate, and high heterogeneity, respectively. Ninety‐five percent prediction intervals were calculated based on the *t*‐distribution to project the expected range of true effects in future similar settings.

The Phase 2b dose‐ranging trial (Ballantyne et al., 2023) contributed four enlicitide dose arms (6, 12, 18, and 30 mg) sharing a single placebo group. To avoid a unit‐of‐analysis error from double‐counting the shared placebo participants, the placebo group was divided into four approximately equal‐sized subgroups distributed across the four pairwise comparisons, with the placebo mean and standard deviation retained and only the sample size apportioned, in accordance with the Cochrane Handbook for Systematic Reviews of Interventions (Higgins et al., section 23.3.4).

The 24‐week sensitivity analysis restricted the pooled estimate to the 20 mg dose at week 24 the common prespecified primary endpoint of the two Phase 3 trials (Navar 2026 and Ballantyne 2025) in order to isolate the clinically and pharmacologically homogeneous data. It is distinct from the treatment‐duration subgroup analysis (8 vs. 52 weeks), which stratifies the full dataset by trial follow‐up; the 24‐week analysis is a homogeneity‐restricted sensitivity check aligning dose and endpoint across the Phase 3 trials, not a third duration stratum.

Because every outcome included substantially fewer than 10 comparisons, formal assessment of small‐study effects and funnel‐plot asymmetry testing was not performed, in line with Cochrane guidance.

Leave‐one‐out sensitivity analyses were performed for all outcomes to highlight the individual arms of the study with disproportionate influence on pooled estimates or heterogeneity. An exploratory subgroup analysis based on the period for treatment (52 and 8 weeks) and drug dosages (6, 12, 18, and 30 mg) was also conducted. All analyses were conducted using R (version 4.5.0) with the ‘meta’ package [25].

## 3 Results

### 3.1 Study Selection and Characteristics

Initial search of the records revealed a total number of 458 studies. After exclusion of 27 duplicate studies, 431 unique studies remained for screening. During the title and abstract review phase of the study, elimination of the majority of studies left 3 articles for full‐text assessment. Finally, 3 randomized controlled trials were included for the study design. The study selection process is illustrated in Figure 1.

Three randomized, double‐blind, placebo‐controlled trials involving 3593 participants met the inclusion criteria. Navar et al. (2026) enrolled 2909 participants across 14 countries and evaluated enlicitide 20 mg once daily over 52 weeks [10]. Ballantyne et al. (2023) randomized 381 participants in an 8‐week dose‐ranging trial of enlicitide 6, 12, 18, and 30 mg once daily [11]. The randomized placebo‐controlled study comprising 303 participants by Ballantyne et al. (2025) was assigned enlicitide 20 mg or placebo over a follow‐up period of 52 weeks [11]. Baseline demographic and clinical characteristics of the included studies are presented in Table 1.

**TABLE 1:** Baseline characteristics of included randomized controlled trials of enlicitide.

Characteristic	Navar et al. 2026 [10]	Ballantyne et al. (2023) [11]	Ballantyne et al. (2025) [12]
Phase	Phase 3	Phase 2b	Phase 3
Design	Multinational, randomized, double‐blind, placebo‐controlled trial	Multicenter, randomized, double‐blind, placebo‐controlled trial	Parallel‐group, randomized, double‐blind, placebo‐controlled trial
Sites/countries	168 sites in 14 countries	63 sites in 8 countries	59 sites across 17 countries
Participants randomized	2909	381	303
Treatment duration	52 weeks	8 weeks	52 weeks
Female, *n* (%)	1142 (39.3)	188 (49.3)	155 (51.2)
Age (Years)	62.8 ± 10.7	61.4 ± 9.7	52.4 ± 13.6
BMI, kg/m^2^	—	29.4 ± 5.8	28.5 ± 5.4
Clinical ASCVD/prior major ASCVD event, *n* (%)	1697 (58.3)	147 (38.6)	81 (26.7)
Type 2 diabetes, *n* (%)	1431 (49.2)	211 (55.4)	37 (12.2)
Any statin use, *n* (%)	2809 (96.6)	231 (60.6)	303 (100.0)
LDL‐C, mg/dL	96.1 ± 38.9	119.5 ± 34.8	119.0 ± 41.0
Non‐HDL‐C, mg/dL	122.8 ± 42.8	149.5 ± 38.8	142.1 ± 45.7
Apolipoprotein B, mg/dL	91.2 ± 26.9	106.0 ± 24.5	103.8 ± 27.6
HDL‐C, mg/dL	50.0 ± 14.0	49.5 ± 14.2	—

*Note:* Data is reported in Mean ± SD.

### 3.2 Meta Analysis Result

#### 3.2.1 Efficacy Outcomes

##### 3.2.1.1 Percentage Change From Baseline in LDL‐C

Across six comparisons from three trials (*n* = 3337), enlicitide was associated with a greater reduction in LDL‐C from baseline than placebo (MD −56.19%; 95% CI −64.14 to −48.25; *p* < 0.0001; 95% prediction interval −72.18% to −40.21%), with moderate heterogeneity (*I* ^2^ = 56.9%) (Figure 2). In the dose‐ranging trial, LDL‐C reduction was numerically greater at higher doses, with the largest reduction observed at 30 mg (MD −60.90%; 95% CI −72.67 to −49.13), although subgroup analysis of the study revealed statistically non‐significant (*p* = 0.12) subgroup difference (Figure S1). Comparison across treatment durations demonstrated no significant variation between the 8 and 52‐week outcomes (*p* = 0.50) (Figure S2). In the 24‐week sensitivity analysis restricted to the 20 mg dose (two comparisons; *n* = 3049), the effect remained consistent (MD −60.39%; 95% CI −64.76 to −56.01; *p* = 0.004; *I* ^2^ = 0%) (Figure S3). Leave‐one‐out analysis did not alter the overall conclusion (Figure S4).

**FIGURE 2:**
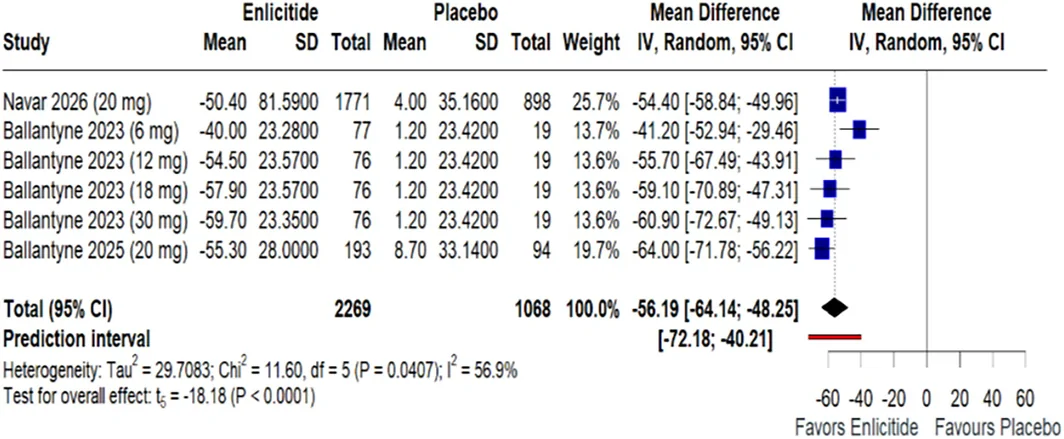
Forest plot of percentage change from baseline in LDL‐C levels.

##### 3.2.1.2 Percentage Change From Baseline in Non‐HDL‐C

Across six comparisons from three trials (*n* = 3338), enlicitide was associated with a reduction in non‐HDL‐C versus placebo (MD −51.36%; 95% CI −58.83 to −43.90; *p* < 0.0001; 95% prediction interval −65.62% to −37.11%), with moderate heterogeneity (*I* ^2^ = 53.8%) (Figure 3). A similar numerical pattern was seen across dose levels, with the largest reduction in the dose‐ranging trial at 30 mg (MD −55.80%); however, subgroup differences were not significant (*p* = 0.11) (Figure S5). There was also no significant difference by treatment duration (*p* = 0.39) (Figure S6). In the 24‐week sensitivity analysis (two comparisons; *n* = 3050), the result remained consistent (MD −56.03%; 95% CI −64.43 to −47.64; *p* = 0.008; *I* ^2^ = 0%) (Figure S7). Leave‐one‐out analysis showed that omission of the 6 mg comparison reduced heterogeneity without changing the direction of effect (Figure S8).

**FIGURE 3:**
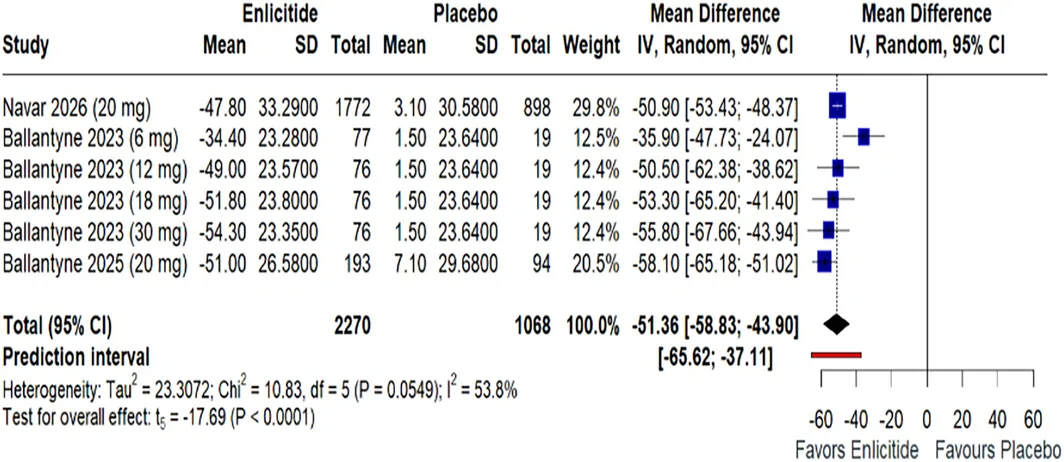
Forest plot of percentage change from baseline in non‐HDL‐C levels.

##### 3.2.1.3 Percentage Change From Baseline in Apolipoprotein‐B

Six comparisons from three trials (*n* = 3325) suggested a greater reduction in ApoB with enlicitide than with placebo (MD −47.09%; 95% CI −53.51 to −40.66; *p* < 0.0001; 95% prediction interval −58.44% to −35.73%), with moderate heterogeneity (*I* ^2^ = 49.8%) (Figure 4). In the dose‐ranging trial, the largest numerical reduction was observed at 30 mg (MD −51.80%), but subgroup differences were not statistically significant (*p* = 0.09) (Figure S9). No significant difference was found between 8 and 52‐week comparisons (*p* = 0.41) (Figure S10). The 24‐week sensitivity analysis yielded a similar estimate (MD −52.15%; 95% CI −63.13 to −41.18; *p* = 0.01; *I* ^2^ = 0%) (Figure S11). Leave‐one‐out analysis did not materially change the overall finding; however, the heterogeneity was reduced by omission of the 6 mg interventional arm (Figure S12).

**FIGURE 4:**
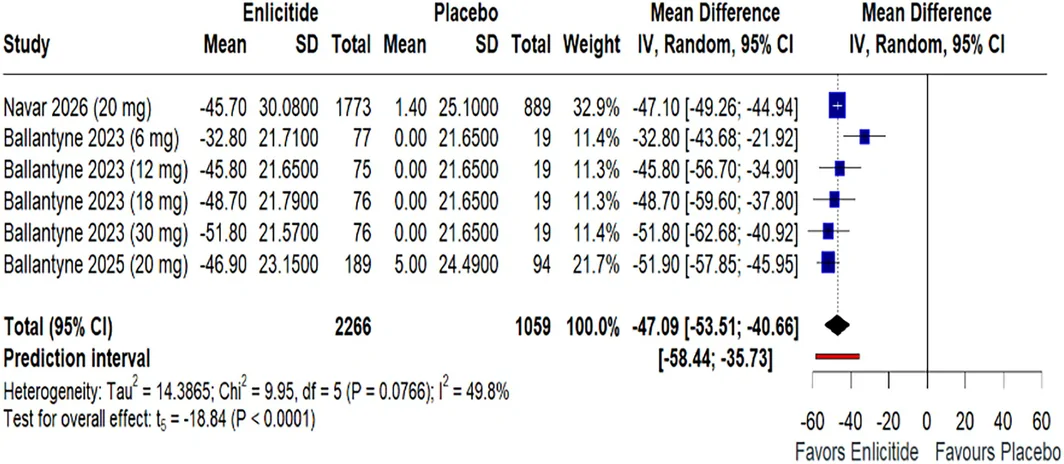
Forest plot of percentage change from baseline in Apolipoprotein‐B levels.

##### 3.2.1.4 Percentage Change From Baseline in Lipoprotein(a)

Across six comparisons from three trials (*n* = 3317), enlicitide significantly reduced lipoprotein(a) compared with placebo (MD −20.10%; 95% CI −25.46 to −14.75; *p* = 0.0002), with no observed heterogeneity (*I* ^2^ = 0%) (Figure 5). Dose subgroup differences were not significant (*p* = 0.10) (Figure S13). In contrast, the treatment‐duration subgroup analysis showed a larger reduction in the 52‐week comparisons than in the 8‐week comparisons (52 weeks: MD −25.96%; 95% CI −27.86 to −24.06; 8 weeks: MD −19.56%; 95% CI −27.83 to −11.29; p for interaction = 0.01) (Figure S14). In the 24‐week sensitivity analysis (two comparisons; *n* = 3067), the estimate was imprecise and did not reach statistical significance under the Hartung‐Knapp model (MD −25.79%; 95% CI −63.12 to 11.55; *p* = 0.07) (Figure S15).

**FIGURE 5:**
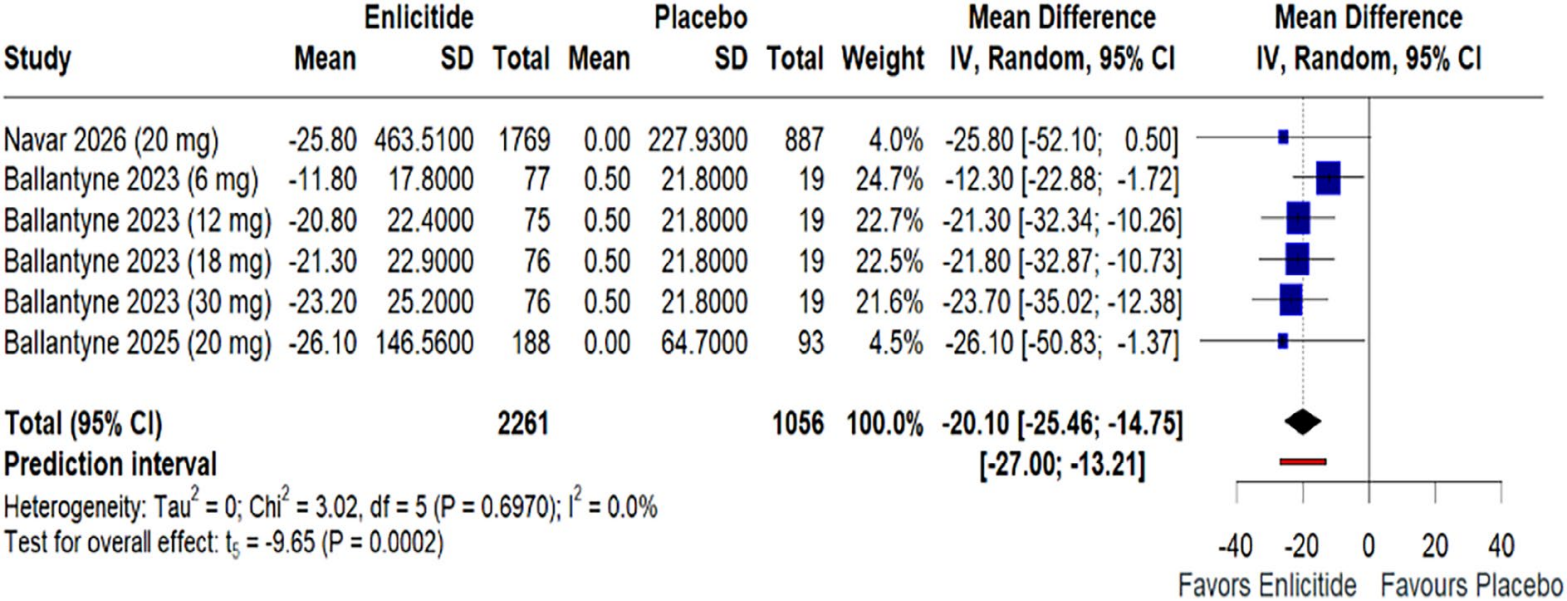
Forest plot of percentage change from baseline in Lipoprotein(a) levels.

##### 3.2.1.5 Percentage Change From Baseline in Triglycerides

Five comparisons from two trials (*n* = 3052) were available, as Ballantyne et al. (2025) did not contribute triglyceride data at the primary analysis time point [12]. Enlicitide was associated with a significant reduction in triglycerides versus placebo (MD −13.12%; 95% CI −17.65 to −8.58; *p* = 0.001), with no heterogeneity (*I* ^2^ = 0%) (Figure 6). The prediction interval crossed the null, suggesting that the magnitude of benefit may be less consistent across settings. No significant difference was observed between 8 and 52‐week comparisons (*p* = 0.95) (Figure S16).

**FIGURE 6:**
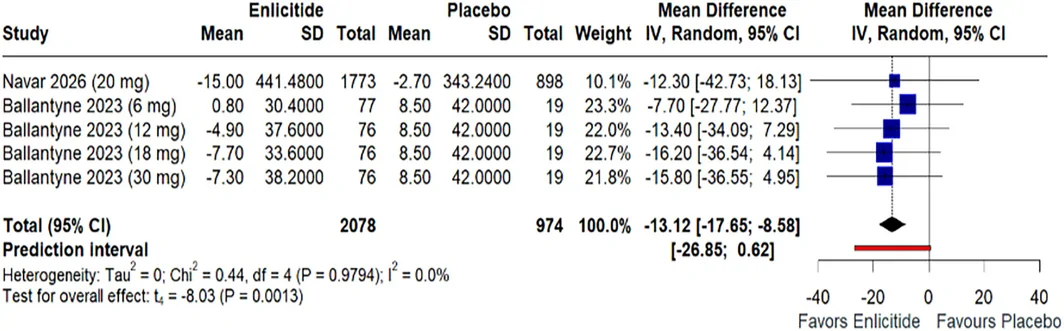
Forest plot of percentage change from baseline in Triglycerides levels.

#### 3.2.2 Safety Outcomes

The safety analysis combined data from all three trials using 24‐week endpoints from Navar 2026 and Ballantyne 2025 with 8‐week endpoints from the four dose arms of Ballantyne 2023 (3588 participants).

##### 3.2.2.1 Any Adverse Event

In the primary analysis (6 comparisons from 3 trials; 2241 events), enlicitide was associated with a statistically significant but clinically marginal increase in the incidence of any adverse event compared with placebo (RR: 1.03; 95% CI: 1.02 to 1.04; *p* = 0.001; I^2^ = 0%). The prediction interval (0.96 to 1.11) crossed the null. Navar 2026 contributed 80.4% of the pooled weight, and the absolute magnitude of the excess risk was small (Figure 7).

**FIGURE 7:**
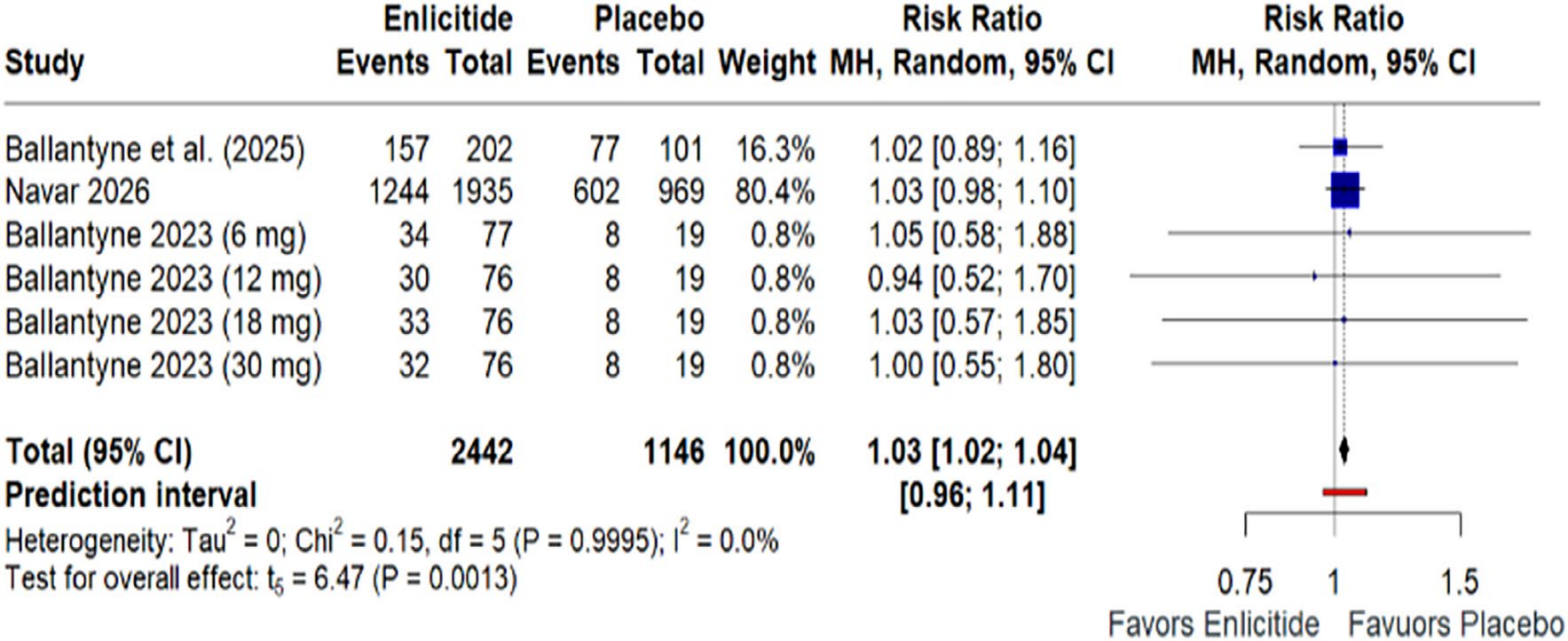
Forest plot of any adverse events.

##### 3.2.2.2 Serious Adverse Events

In the primary analysis (6 comparisons from 3 trials; 328 events), enlicitide was associated with a statistically significant reduction in serious adverse events (RR: 0.84; 95% CI: 0.71 to 1.00; *p* = 0.048; *I* ^2^ = 0%) (Figure 8). However, the prediction interval (0.64 to 1.12) crossed the null, and Navar 2026 contributed 95.7% of the pooled weight, indicating that this result is heavily driven by a single trial. Treatment arm continuity correction was applied for dose arms with zero events. Given the borderline *p*‐value and prediction interval crossing 1.0, this finding should be interpreted with caution pending confirmation from larger cardiovascular outcome trials.

**FIGURE 8:**
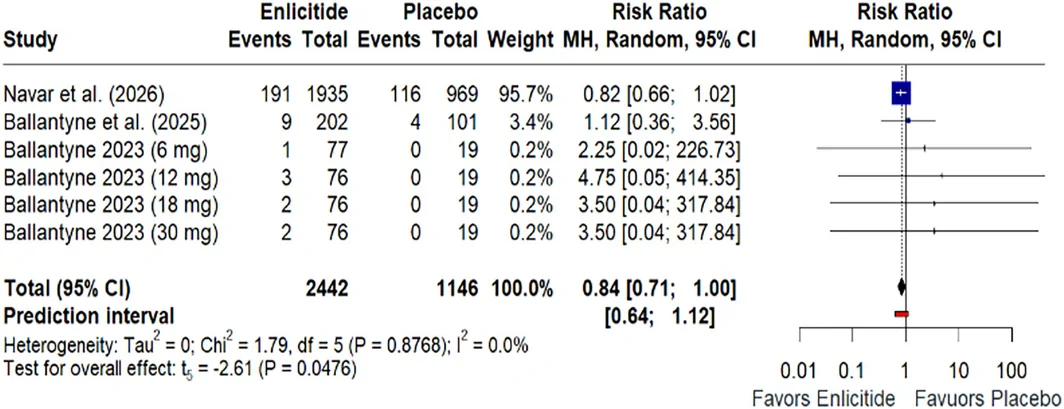
Forest plot of serious adverse events.

##### 3.2.2.3 Moderate‐To‐Severe Adverse Events

In the primary analysis (6 comparisons from 3 trials; 1111 events), no significant difference was observed between groups (RR: 1.02; 95% CI: 0.97 to 1.08; *p* = 0.38; *I* ^2^ = 0%). The prediction interval (0.89 to 1.17) crossed the null (Figure 9).

**FIGURE 9:**
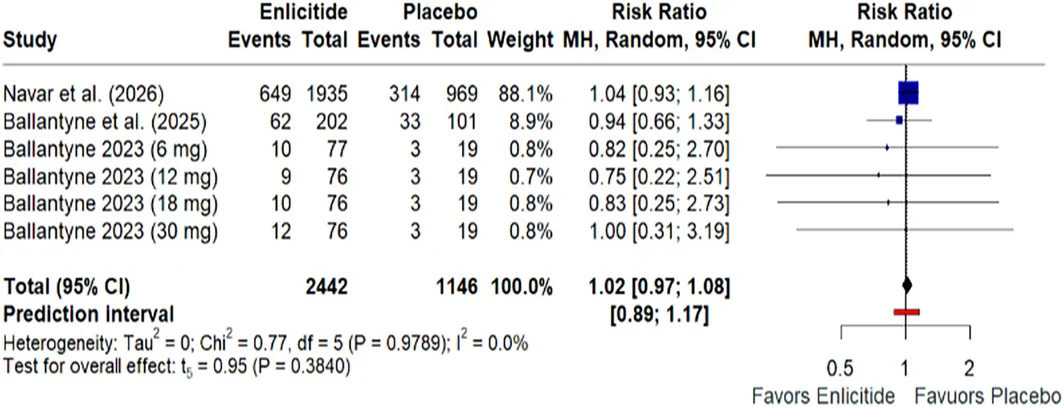
Forest plot of moderate‐to‐severe adverse events.

##### 3.2.2.4 Treatment Discontinuation due to Adverse Events

In the primary analysis (5 comparisons from 3 trials; 113 events), there was a non‐significant trend towards fewer discontinuations due to adverse events in the enlicitide group (RR: 0.77; 95% CI: 0.57 to 1.05; *p* = 0.08; *I* ^2^ = 0%) (Figure 10). The prediction interval (0.45 to 1.31) was wide and crossed the null. Navar 2026 contributed 91.5% of the pooled weight. Treatment arm continuity correction was applied for one dose arm with zero events.

**FIGURE 10:**
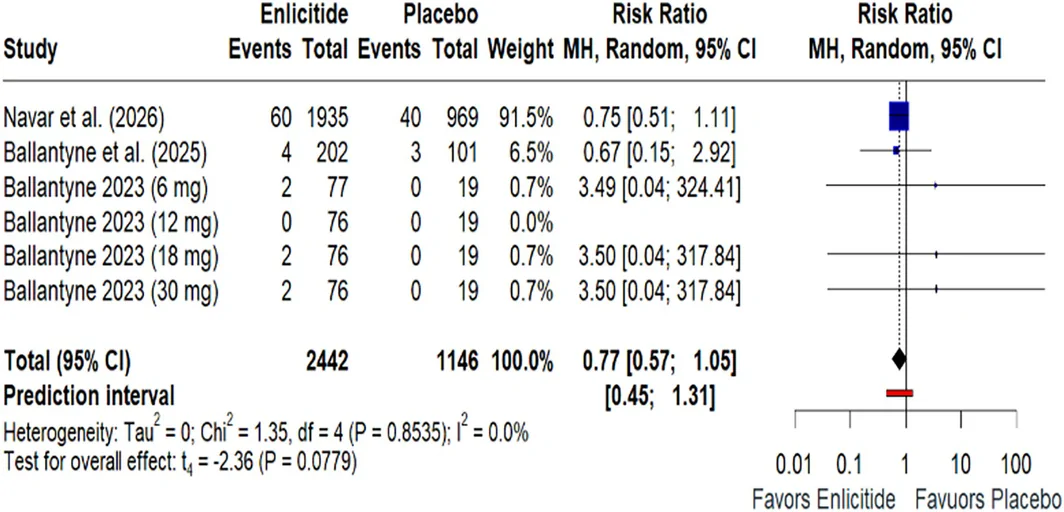
Forest plot of treatment discontinuation due to adverse events.

##### 3.2.2.5 All‐Cause Mortality

In the primary analysis (3 comparisons from 3 trials; 22 events), no significant difference in mortality was observed (RR: 1.01; 95% CI: 0.44 to 2.33; *p* = 0.95; *I* ^2^ = 0%) (Figure 11). The prediction interval (0.15 to 6.89) was extremely wide. Treatment arm continuity correction was applied.

**FIGURE 11:**
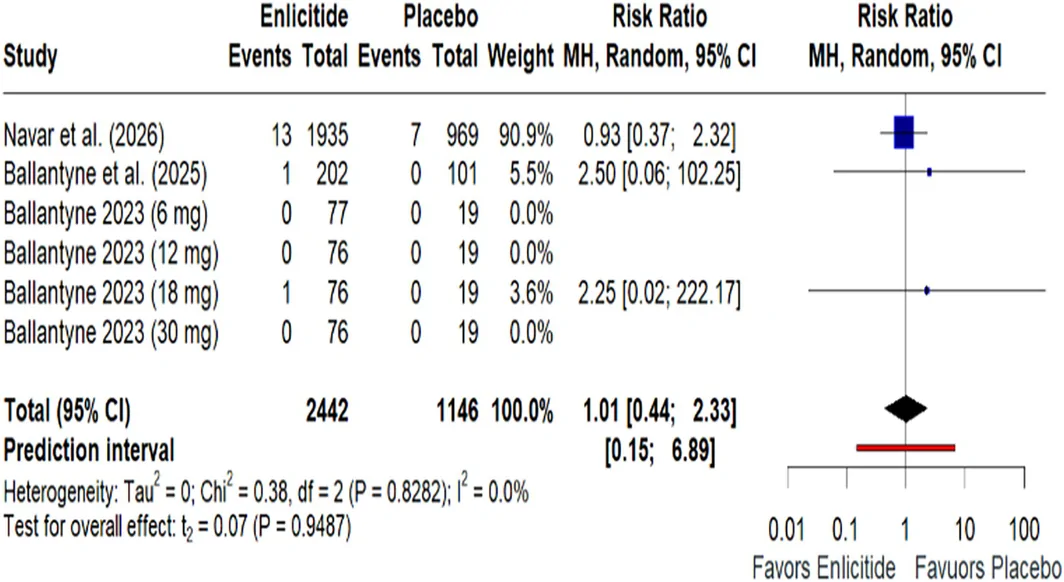
Forest plot of all‐cause mortality.

#### 3.2.3 Risk of Bias Assessment

The detailed RoB 2.0 assessment of the three included RCTs showed an overall low risk of bias in two trials and some concerns in one trial, with no trial judged to be at high risk of bias. The main reason for downgrading to some concerns was related to the domain of bias arising from the randomization process and selection of reported results. Overall, the included trials were considered to be of acceptable methodological quality, although these concerns reduced confidence in some pooled estimates. The risk‐of‐bias traffic light plot is displayed in Figure 12, and the risk‐of‐bias summary plot is shown in Figure S17.

**FIGURE 12:**
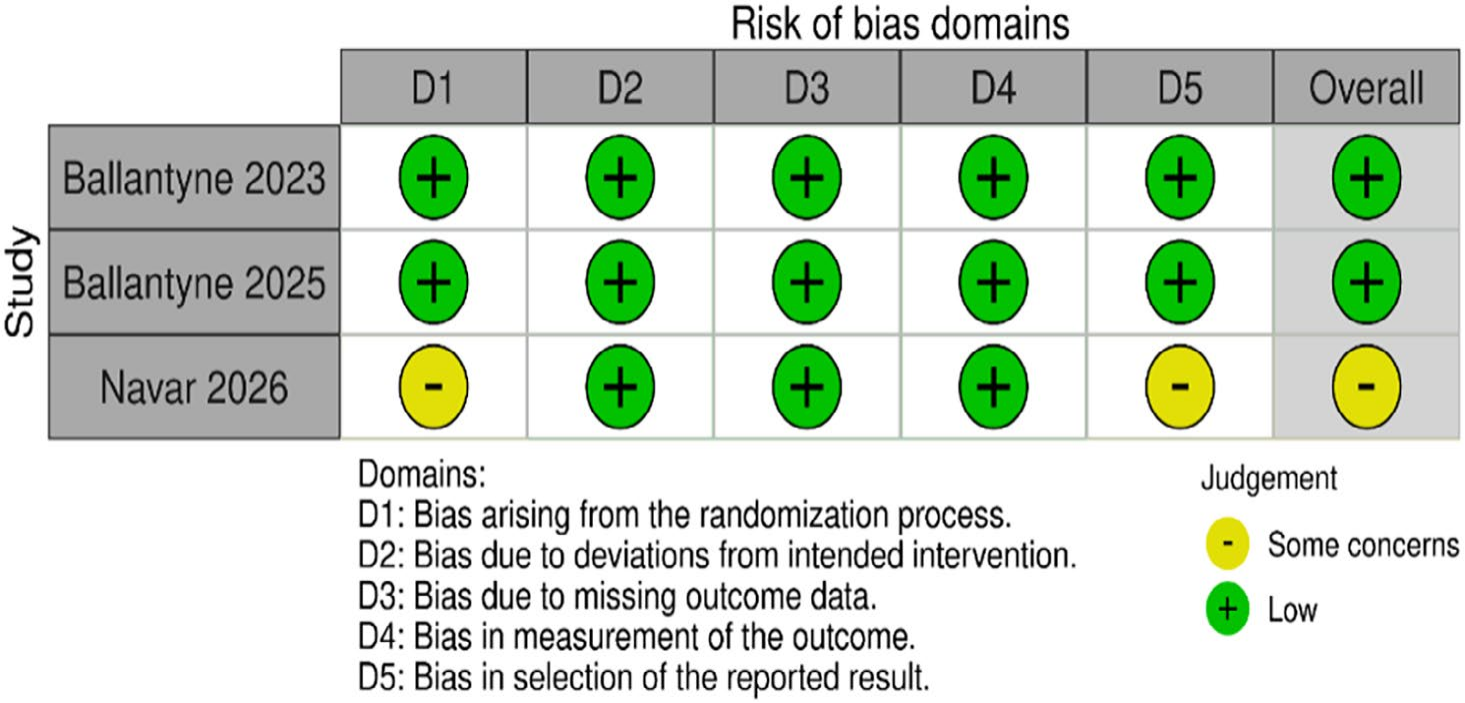
Risk‐of‐bias assessment for the randomized controlled trials included in this meta‐analysis.

#### 3.2.4 Certainty Assessment

The certainty of evidence ranged from moderate to very low across the assessed outcomes. Moderate‐certainty evidence was observed for the LDL‐C, non‐HDL‐C, Apolipoprotein‐B, Lipoprotein(a) and triglycerides change. Low certainty of evidence was observed for any adverse events. Moderate to severe adverse events and discontinuation due to adverse event were having very low certainty while serious adverse events and all‐cause mortality were also marked as very low. Downgrading was most commonly driven by indirectness across the included outcomes, along with some concerns in trial quality and imprecision in several outcomes. The detailed GRADE Summary of findings table is presented in Table S4.

## 4 Discussion

This systematic review and meta‐analysis integrates the findings of three randomized, double‐blind, placebo‐controlled trials, including 3593 participants, to evaluate the clinical efficacy and safety profile of enlicitide [10, 11, 12]. Pooling these trials allowed for a more precise assessment of the magnitude and consistency of the lipid‐lowering effects of enlicitide across Phase 2 b and Phase 3 settings, multiple dose levels, and distinct patient populations. However, because all included trials were based on surrogate lipid endpoints, these findings support lipid‐lowering efficacy rather than definitive long‐term clinical benefit [8, 10, 11, 12, 15].

This question is clinically relevant because many patients with high cardiovascular stress remain above the recommended thresholds of LDL‐C levels despite currently available therapies, and contemporary guidelines recommend stepwise intensification with non‐statin agents when statins alone are insufficient [26, 27, 28]. Injectable PCSK9‐directed therapies have shown that deeper LDL‐C lowering can translate into cardiovascular benefit, but constrained implication of intervention in hospital settings highlights the limited accessibility, prior authorization, cost consideration, and patient reluctance towards parenteral administration [4, 5, 29, 30]. In order to address the existing therapeutic gap, an orally administered PCSK9 inhibitor such as enlicitide is introduced as a clinically promising and potentially acceptable alternative to injectable PCSK9 inhibitors and other known lipid‐lowering therapies [29, 30]. Pharmacologically, enlicitide increases the clearance of circulating LDL particles by preventing the interaction of PCSK9 molecules from the bloodstream with LDL receptors of the body [9]. This places enlicitide in the same mechanistic pathway as injectable monoclonal antibody PCSK9 inhibitors and helps explain why the LDL‐C reductions observed in these trials approach the range reported for established injectable agents [4, 5, 9].

Most importantly, the extended benefits of enlicitide along with reduced levels of LDL‐C further strengthen the clinical utility of the drug. Significantly improved levels of LDL‐C, non–HDL‐C, and ApoB evident from the dose–response phase of the study explain the secondary objectives of our manuscript [10]. In our pooled analysis, a numerical dose–response gradient remained visible across these outcomes, with larger effects at higher doses, although subgroup interaction testing was not statistically significant. Contemporary evidence signifies the clinical relevance of biomarkers like apoB and non‐HDL‐C as a better reflection of cardiovascular risk than LDL‐C alone. However, the limited amount of available data warrants the cautious interpretation of the observed findings.

These findings also align with the broader emerging literature on oral PCSK9 inhibition. Recently pooled data from early‐phase trials Masson et al. reported substantially reduced levels of LDL‐C approximately 48% alongside meaningful decreases in ApoB and Lp(a) without a clear excess of adverse events compared with placebo [8]. The magnitude of LDL‐C reduction in our analysis, which includes larger Phase 3 datasets, was numerically greater, likely reflecting the use of the optimized 20 mg dose in the Phase 3 trials rather than pooling across multiple dose levels. Our findings therefore extend and refine those earlier estimates while confirming the consistency of the class‐level signal [8, 31]. A recent class‐level network meta‐analysis of oral PCSK9 inhibitors (Kamrul‐Hasan et al. Endocrine Practice 2026) [14] pooled four RCTs (*N* = 1387) with a literature search concluding on 14 November 2025. Our study differs in three substantive respects: it is agent‐specific, focusing exclusively on enlicitide and thereby avoiding the transitivity and cross‐agent assumptions inherent to a network analysis; our search extends through March 2026 and therefore incorporates the large Phase 3 enlicitide dataset that postdates the earlier search window, yielding an enlicitide‐specific evidence base larger than the total across all agents in the prior review; and we provide analyses not available in the class‐level approach, including a formal dose–response evaluation across the enlicitide dose range, treatment‐duration subgroup analyses, outcome‐level GRADE certainty ratings, and pooled estimates for lipoprotein(a) and triglycerides. The oral PCSK9 inhibitor landscape is also broadening beyond enlicitide; the PURSUIT trial of AZD0780, a structurally distinct small‐molecule oral PCSK9 inhibitor, has similarly demonstrated dose‐dependent LDL‐C lowering and acceptable short‐term tolerability, and several other oral PCSK9‐targeting strategies are in earlier stages of clinical development [6, 13]. Although the exclusion of CORALreef AddOn trials from the present pooled analysis, which was restricted to placebo‐controlled studies, it provides useful context for the likely clinical positioning of enlicitide among oral non‐statin options [32].

The apparent benefit was not confined to LDL‐C alone. Parallel reductions in Lp(a), non–HDLC, and ApoB suggest improvement across the broader burden of atherogenic lipoproteins rather than in a single laboratory marker [8, 10, 11, 12, 33]. For each of these core outcomes, the 95% prediction interval excluded the null, indicating that a clinically meaningful effect would be expected in a future similar trial setting. These multi‐marker reductions are clinically noteworthy because emerging evidence supports ApoB and non–HDL‐C as complementary treatment targets that may capture residual atherogenic risk not fully reflected by LDL‐C alone [27, 33]. The reduction in lipoprotein(a) is also notable because prior studies of PCSK9 inhibition suggest that Lp(a) lowering may contribute to cardiovascular risk reduction beyond LDL‐C alone [34, 35]. In our subgroup analyses, Lp(a) was the only outcome to show a duration‐related difference, with larger reductions in the 52‐week comparisons than in the 8‐week comparisons, raising the possibility that this effect becomes more apparent with longer exposure. However, this finding should be viewed as exploratory because the evidence base for Lp(a) remains less mature than that for LDL‐C, ApoB, and non–HDL‐C [34, 35]. In the CORALreef Lipids trial, over 70% of the included participants achieved reduced levels of LDL‐C (< 70 mg/dL) with at least a 50% reduction from baseline, and approximately two‐thirds of the population reached the more stringent target of below 55 mg/dL [11]. Although these goal‐attainment rates were reported in individual trials rather than derived from our pooled analysis, the consistency of the pooled effect sizes across studies suggests that similarly high rates of goal attainment would be expected in analogous populations, a consideration that may inform guideline‐directed therapeutic decision‐making [7, 27].

Some indirect cross‐trial comparisons may suggest LDL‐C reductions with enlicitide that are numerically similar to or greater than those reported with inclisiran, but such cross‐trial comparisons should be interpreted cautiously because study populations, background therapy, follow‐up duration, and trial designs differ [4, 5, 36, 37]. The main practical advantage of enlicitide is therefore not proven superiority over injectable agents, but the possibility of delivering clinically meaningful LDL‐C reduction through an oral route [3, 33]. That advantage should also not be overstated. In the Phase 3 trials, participants were instructed to take enlicitide on an empty stomach and avoid food and beverages other than water for 30 min afterward, which may affect adherence outside trial settings [7, 11]. Reassuringly, however, adherence to both the study drug and fasting instructions exceeded 97% in the CORALreef Lipids trial, suggesting that the regimen was practical for most participants under controlled conditions [11, 31]. Whether this level of adherence can be maintained in routine clinical practice, where patient populations are more heterogeneous and follow‐up is less structured, remains to be established.

The safety findings should also be interpreted with balance. The pooled analysis of included placebo‐controlled trials did not demonstrate any statistically significant differences between enlicitide and placebo in terms of overall adverse events, serious adverse events, or treatment discontinuation. Notably, new‐onset or worsening diabetes mellitus, a prespecified event of clinical interest in the Phase 3 trials, occurred at comparable rates between enlicitide and placebo groups, and no cases of drug‐induced liver injury were reported [11, 12, 32]. These observations are consistent with the broader safety record of the PCSK9 inhibitor class, in which large cardiovascular outcomes trials of injectable agents have similarly not identified excess diabetes risk or hepatotoxicity [4, 5, 37]. Overall, the current evidence supports acceptable short‐term tolerability, but the dataset remains small, follow‐up does not extend beyond 52 weeks, and uncommon or delayed harms cannot yet be excluded.

This meta‐analysis has several methodological strengths. According to the established standards of reporting, our study design exhibits prominent statistical approaches including a random‐effects model with conservative adjustments. This manuscript provides the complete evaluation of both the certainty of evidence and the risk of bias. Moreover, the subgroup analysis of the study highlights the robustness of the observed findings and potential sources of heterogeneity. Inclusion of the trials with low risk of bias further strengthens the validity of the clinical interpretations from the manuscript.

## 5 Limitation

Several limitations should be acknowledged. The evidence base remains small, some pooled comparisons were derived from multiple dose arms within the same Phase 2b trial, and the literature is still dominated by surrogate lipid endpoints. The limited follow‐up period of the study (52 weeks) fails to explain the durability of the benefit and late adverse effects of the intervention [8, 33]. All included trials were industry‐sponsored, which is expected for investigational agents but should be considered when interpreting the totality of the evidence. Because every outcome included substantially fewer than 10 comparisons, formal assessment of small‐study effects and funnel‐plot asymmetry testing was not performed, in line with Cochrane guidance. Additionally, individual patient data were not available, which precluded more granular analyses of treatment‐effect modification by baseline LDL‐C level, statin intensity, or comorbid conditions. The generalizability of the findings may also be limited by the underrepresentation of certain demographic and geographic subgroups in the included trials. These limitations do not negate the central lipid‐lowering signal, but they do define the level of certainty that can reasonably be claimed at this stage [8, 10, 11, 12].

## 6 Future Direction

The reduced frequency of cardiovascular events with the lipid‐lowering effect of enlicitide remains unanswered. Inclusion of large‐scale outcome trials in future studies is necessary to evaluate the complete efficacy profile of this novel intervention. The broader trajectory of PCSK9 inhibitor development—in which injectable agents first demonstrated robust LDL‐C lowering in surrogate‐endpoint trials and subsequently proved cardiovascular benefit in large outcomes studies—provides a plausible framework for anticipating the trajectory of oral PCSK9 inhibitors, although such inferences remain indirect [4, 5, 37]. Future trials should further clarify the role of enlicitide through adequately powered cardiovascular outcomes studies, longer‐term follow‐up, and active‐comparator designs against established non‐statin therapies. They should also enrol broader and underrepresented high‐risk populations, apply standardized efficacy and safety endpoints, and evaluate adherence, persistence, and feasibility under real‐world dosing conditions. Until such evidence matures, enlicitide should be regarded as a promising oral LDL‐lowering agent with strong surrogate efficacy and acceptable short‐term tolerability. Meanwhile, the real‐world implication of the intervention depends on the long‐term safety, comparative effectiveness, pricing, access, and real‐world adherence of the intervention [9, 15, 27, 28, 29, 30].

## 7 Conclusion

In conclusion, this meta‐analysis suggests that enlicitide is a promising oral LDL‐lowering agent in development. Its lipid‐lowering effect appears reasonably consistent across the three currently available randomized placebo‐controlled trials, and its short‐term tolerability appears acceptable. These findings should be interpreted cautiously in light of the small number of trials, moderate heterogeneity in several efficacy outcomes, and the fact that several subgroup and secondary findings including the dose–response gradient, the duration‐related lipoprotein(a) effect, and the triglyceride estimate are exploratory and substantially driven by single trials. The long‐term clinical value of enlicitide will ultimately depend on whether these lipid improvements translate into durable cardiovascular benefit in adequately powered outcomes trials, and whether the practical advantages of oral administration are preserved in routine care.

## Author Contributions

All authors contributed to the study conception and design. Muhammad Farhan and Muhammad Murtaza conceptualized the topic. Muhammad Farhan, Muhammad Murtaza, and Muhammad Mujtaba Rasool performed the literature search and managed study selection. Title and abstract writing were done by Ahmed Asad Raza. Hammad Amjad, Farah Khan, and Muhammad Murtaza completed full text screening and data extraction. Data analysis was done by Muhammad Mujtaba Rasool. Kuldeep Dalpat Rai, Farah Khan, Hammad Amjad, and Abedin Samadi wrote the manuscript. Revisions and supervision were provided by Sheikh Muhammad Ebtehaj Ali, Muhammad Farhan, Furqan Hassan, and Muhammad Ahmad Sohail. All authors read and approved of the final manuscript.

## Funding

The authors have nothing to report.

## Disclosure

The authors declare that all data were generated in‐house, that no paper mill was used, and that no AI tool has been used for the generation of text or figures.

## Ethics Statement

The authors have nothing to report.

## Consent

All authors have reviewed the final draft and agreed to the publication of the manuscript in its current form.

## Conflicts of Interest

The authors declare no conflicts of interest.

## Supporting information


**Table S1:** PRISMA 2020 Main Checklist.
**Table S2:** PRISMA Abstract Checklist.
**Table S3:** Search Strategy.
**Table S4:** Grade Assessment.
**Figure S1:** Forest plot of subgroup analysis stratified by dose for percentage change from baseline in LDL‐C.
**Figure S2:** Forest plot of subgroup analysis stratified by treatment durations for percentage change from baseline in LDL‐C.
**Figure S3:** Sensitivity analysis for percentage change from baseline in LDL‐C.
**Figure S4:** Leave‐one‐out analysis for percentage change from baseline in LDL‐C.
**Figure S5:** Forest plot of subgroup analysis stratified by dose for percentage change from baseline in Non‐HDL‐C.
**Figure S6:** Forest plot of subgroup analysis stratified by treatment durations for percentage change from baseline in Non‐HDL‐C.
**Figure S7:** Sensitivity analysis for percentage change from baseline in Non‐LDL‐C.
**Figure S8:** Leave‐one‐out analysis for percentage change from baseline in Non‐LDL‐C.
**Figure S9:** Forest plot of subgroup analysis stratified by dose for percentage change from baseline in ApoB.
**Figure S10:** Forest plot of subgroup analysis stratified by treatment durations for percentage change from baseline in ApoB.
**Figure S11:** Sensitivity analysis for percentage change from baseline in ApoB.
**Figure S12:** Leave‐one‐out analysis for percentage change from baseline in ApoB.
**Figure S13:** Forest plot of subgroup analysis stratified by dose for percentage change from baseline in Lipoprotein(a).
**Figure S14:** Forest plot of subgroup analysis stratified by treatment durations for percentage change from baseline in Lipoprotein(a).
**Figure S15:** Sensitivity analysis for percentage change from baseline in Lipoprotein(a).
**Figure S16:** Forest plot of subgroup analysis stratified by treatment durations for percentage change from baseline in Triglycerides.
**Figure S17:** Risk of Bias Summary Plot.

## Data Availability

All data generated or analysed during this study are included in the manuscript and its Supporting Information.

## References

[1] M. B. Mortensen , O. Dzaye , H. E. Bøtker , et al., “Low‐Density Lipoprotein Cholesterol Is Predominantly Associated With Atherosclerotic Cardiovascular Disease Events in Patients With Evidence of Coronary Atherosclerosis: The Western Denmark Heart Registry,” Circulation 147, no. 14 (2023): 1053–1063, 10.1161/CIRCULATIONAHA.122.061010. PMC10073288 36621817

[2] O. Mhaimeed , Z. A. Burney , S. L. Schott , P. Kohli , F. A. Marvel , and S. S. Martin , “The Importance of LDL‐C Lowering in Atherosclerotic Cardiovascular Disease Prevention: Lower for Longer Is Better,” American Journal of Preventive Cardiology 18 (2024): 100649, 10.1016/j.ajpc.2024.100649. PMC10992711 38576462

[3] C. M. Ballantyne , A. L. Catapano , and K. Nasir , “Oral PCSK9 Inhibitors: Can Novel Therapies Overcome Barriers to Effective LDL‐Cholesterol Management?,” Journal of the American College of Cardiology 85, no. 21 (2025): 2008–2010, 10.1016/j.jacc.2025.03.518. 40304643

[4] M. S. Sabatine , R. P. Giugliano , A. C. Keech , et al., “Evolocumab and Clinical Outcomes in Patients With Cardiovascular Disease,” New England Journal of Medicine 376, no. 18 (2017): 1713–1722, 10.1056/NEJMoa1615664. 28304224

[5] G. G. Schwartz , P. G. Steg , M. Szarek , et al., “Alirocumab and Cardiovascular Outcomes After Acute Coronary Syndrome,” New England Journal of Medicine 379, no. 22 (2018): 2097–2107, 10.1056/NEJMoa1801174. 30403574

[6] N. Ferri and G. Marodin , “Emerging Oral Therapeutic Strategies for Inhibiting PCSK9,” Atheroscler Plus 59 (2025): 25–31, 10.1016/j.athplu.2024.11.003. PMC11722601 39802651

[7] W. E. Boden , “Exploring a New “Reef” in Dyslipidemic Risk Reduction,” New England Journal of Medicine 394, no. 6 (2026): 597–599, 10.1056/NEJMe2516860. 41885976

[8] W. Masson , M. Lobo , G. Giunta , L. Barbagelata , and J. P. Nogueira , “Lipid‐Lowering Efficacy and Safety of Oral Proprotein Convertase Subtilisin/Kexin Type 9 Inhibitors: A Systematic Review and Meta‐Analysis,” Advances in Therapy 43 (2026): 304–316, 10.1007/s12325-025-03418-x. PMC12858574 41288928

[9] D. G. Johns , L. C. Campeau , P. Banka , et al., “Orally Bioavailable Macrocyclic Peptide That Inhibits Binding of PCSK9 to the Low‐Density Lipoprotein Receptor,” Circulation 148, no. 2 (2023): 144–158, 10.1161/CIRCULATIONAHA.122.063372. PMC10325562 37125593

[10] C. M. Ballantyne , P. Banka , G. Mendez , et al., “Phase 2b Randomized Trial of the Oral PCSK9 Inhibitor MK‐0616,” Journal of the American College of Cardiology 81, no. 16 (2023): 1553–1564, 10.1016/j.jacc.2023.02.018. 36889610

[11] A. M. Navar , E. Mikhailova , A. L. Catapano , et al., “A Placebo‐Controlled Trial of the Oral PCSK9 Inhibitor Enlicitide,” New England Journal of Medicine 394, no. 6 (2026): 529–539, 10.1056/NEJMoa2511002. 41879224

[12] C. M. Ballantyne , L. Gellis , J. C. Tardif , et al., “Efficacy and Safety of Oral PCSK9 Inhibitor Enlicitide in Adults With Heterozygous Familial Hypercholesterolemia: A Randomized Clinical Trial,” Journal of the American Medical Association 335, no. 2 (2026): 129–139, 10.1001/jama.2025.20620. PMC12598580 41206969

[13] M. J. Koren , R. B. Vega , N. Agrawal , et al., “An Oral PCSK9 Inhibitor for Treatment of Hypercholesterolemia: The PURSUIT Randomized Trial,” Journal of the American College of Cardiology 85, no. 21 (2025): 1996–2007, 10.1016/j.jacc.2025.03.499. 40167413

[14] A. B. M. Kamrul‐Hasan , K. Mahajan , I. Khalil , et al., “Safety and Efficacy of Oral PCSK9 Inhibitors in Hypercholesterolemia: A Systematic Review and Network Meta‐Analysis,” Endocrine Practice : Official Journal of the American College of Endocrinology and the American Association of Clinical Endocrinologists 32, no. 6 (2026): 962–971, 10.1016/j.eprac.2026.02.002. 41690582

[15] “ClinicalTrials.gov. Enlicitide Decanoate (MK‐0616 oral PCSK9 inhibitor) cardiovascular outcomes study (CORALreef Outcomes),” Identifier: NCT06008756, accessed April 9, 2026.

[16] M. J. Page , J. E. McKenzie , P. M. Bossuyt , et al., “The PRISMA 2020 Statement: An Updated Guideline for Reporting Systematic Reviews,” PLoS Medicine 18, no. 3 (2021): e1003583, 10.1371/journal.pmed.1003583. PMC8007028 33780438

[17] PROSPERO , “International Prospective Register of Systematic Reviews,” accessed April 5, 2026, https://www.crd.york.ac.uk/PROSPERO/view/CRD420261357317.

[18] M. Prasad , “Introduction to the GRADE Tool for Rating Certainty in Evidence and Recommendations,” Clinical Epidemiology and Global Health 25 (2024): 101484, 10.1016/j.cegh.2023.101484.

[19] J. P. T. Higgins , D. G. Altman , P. C. Gøtzsche , et al., “The Cochrane Collaboration's Tool for Assessing Risk of Bias in Randomized Trials,” BMJ 343 (2011): d5928, 10.1136/bmj.d5928. PMC3196245 22008217

[20] W. Viechtbauer , “Bias and Efficiency of Meta‐Analytic Variance Estimators in the Random‐Effects Model,” Journal of Educational and Behavioral Statistics 30, no. 3 (2005): 261–293, 10.3102/10769986030003261.

[21] J. J. Deeks , J. P. T. Higgins , and D. G. Altman , “Chapter 10: Analysing Data and Undertaking Meta‐Analyses,” in Cochrane Handbook for Systematic Reviews of Interventions. Version 6.5, ed. J. P. T. Higgins , J. Thomas , J. Chandler , et al. (Cochrane, 2024).

[22] J. Hartung and G. Knapp , “A Refined Method for the Meta‐Analysis of Controlled Clinical Trials With Binary Outcome,” Statistics in Medicine 20, no. 24 (2001): 3875–3889, 10.1002/sim.1009. 11782040

[23] J. Inthout , J. P. A. Ioannidis , and G. F. Borm , “The Hartung‐Knapp‐Sidik‐Jonkman Method for Random‐Effects Meta‐Analysis Is Straightforward and Considerably Outperforms the Standard DerSimonian‐Laird Method,” BMC Medical Research Methodology 14 (2014): 25, 10.1186/1471-2288-14-25. PMC4015721 24548571

[24] J. P. T. Higgins , S. G. Thompson , J. J. Deeks , and D. G. Altman , “Measuring Inconsistency in Meta‐Analyses,” BMJ 327, no. 7414 (2003): 557–560, 10.1136/bmj.327.7414.557. PMC192859 12958120

[25] S. Balduzzi , G. Rücker , and G. Schwarzer , “How to Perform a Meta‐Analysis With R: A Practical Tutorial,” Evidence‐Based Mental Health 22, no. 4 (2019): 153–160, 10.1136/ebmental-2019-300117. PMC10231495 31563865

[26] D. M. Lloyd‐Jones , P. B. Morris , C. M. Ballantyne , et al., “ACC Expert Consensus Decision Pathway on the Role of Nonstatin Therapies for LDL Cholesterol Lowering in the Management of Atherosclerotic Cardiovascular Disease Risk,” Journal of the American College of Cardiology 80, no. 14 (2022): 1366–1418, 10.1016/j.jacc.2022.07.006. 36031461

[27] F. Mach , K. C. Koskinas , J. E. R. van Lennep , et al., “Focused Update of the 2019 ESC/EAS Guidelines for the Management of Dyslipidaemias,” European Heart Journal 46, no. 42 (2025): 4359–4378, 10.1093/eurheartj/ehaf190. 40878289

[28] K. K. Ray , I. Haq , A. Bilitou , et al., “Treatment Gaps in LDL Cholesterol Control Among High‐ and Very High‐Risk Patients in Europe: The SANTORINI Study,” Lancet Regional Health ‐ Europe 29 (2023): 100624, 10.1016/j.lanepe.2023.100624. PMC10119631 37090089

[29] A. M. Navar , B. Taylor , H. Mulder , et al., “Association of Prior Authorization and Out‐Of‐Pocket Costs With Patient Access to PCSK9 Inhibitor Therapy,” JAMA Cardiology 2, no. 11 (2017): 1217–1225, 10.1001/jamacardio.2017.3451. PMC5963012 28973087

[30] D. E. MacDougall , S. J. Baum , C. D. Ahmed , M. P. McGowan , and K. A. Wilemon , “Trends in Patient Access to and Utilization of Prescribed PCSK9 Inhibitors in a Large US Claims Database From 2015 to 2021,” Circulation. Cardiovascular Quality and Outcomes 17, no. 2 (2024): e009988, 10.1161/CIRCOUTCOMES.123.009988. PMC10880926 38362767

[31] I. Müller‐Kozarez and U. Laufs , “Phase 3 Study Results for Oral PCSK9 Inhibition With Enlicitide,” Medicus 7, no. 4 (2026): 101097, 10.1016/j.medj.2026.101097. 41966715

[32] A. L. Catapano , E. Mikhailova , A. M. Navar , et al., “Oral PCSK9 Inhibitor Enlicitide Versus Oral Nonstatin Therapies: A Phase 3 Randomized Clinical Trial,” Journal of the American College of Cardiology 88, no. 3 (2026): 340–352, 10.1016/j.jacc.2026.03.036. 42017875

[33] C. M. Ballantyne and G. D. Norata , “The Evolving Landscape of Targets for Lipid Lowering: From Molecular Mechanisms to Translational Implications,” European Heart Journal 46, no. 44 (2025): 4737–4750, 10.1093/eurheartj/ehaf606. 40911366

[34] M. L. O'Donoghue , S. Fazio , R. P. Giugliano , et al., “Lipoprotein(a), PCSK9 Inhibition, and Cardiovascular Risk,” Circulation 139, no. 12 (2019): 1483–1492, 10.1161/CIRCULATIONAHA.118.037184. 30586750

[35] G. G. Schwartz , M. Szarek , V. A. Bittner , et al., “Lipoprotein(a) and Benefit of PCSK9 Inhibition in Patients With Nominally Controlled LDL Cholesterol,” Journal of the American College of Cardiology 78, no. 5 (2021): 421–433, 10.1016/j.jacc.2021.04.102. PMC8822604 34325831

[36] K. K. Ray , R. S. Wright , D. Kallend , et al., “Two Phase 3 Trials of Inclisiran in Patients With Elevated LDL Cholesterol,” New England Journal of Medicine 382, no. 16 (2020): 1507–1519, 10.1056/NEJMoa1912387. 32187462

[37] M. S. Sabatine and U. Laufs , “Proprotein Convertase Subtilisin/Kexin Type 9 Inhibitors: Past, Present, and Future,” European Heart Journal 47, no. 26 (2026): 3366–3374, 10.1093/eurheartj/ehag148. 41841775

