# Supplementary material for: Efficacy and Safety of Enlicitide in Hypercholesterolemia: A Systematic Review and Meta‐Analysis of Randomized Controlled Trials
Source: Endocrinol Diabetes Metab. 2026 Oct 13;9(6):e70358. doi: 10.1002/edm2.70358 (PMC13659930; doi:10.1002/edm2.70358)
Supplement: Supplementary file 1 [file EDM2-9-e70358-s001.docx]

**SUPPLEMENTARY MATERIAL**

**Table S1:** *PRISMA 2020 Main Checklist*

| **Topic** | **No.** | **Item** | **Location where item is reported** |
| --- | --- | --- | --- |
| **TITLE** |  |  |  |
| **Title** | 1 | Identify the report as a systematic review. | 1 |
| **ABSTRACT** |  |  |  |
| **Abstract** | 2 | See the PRISMA 2020 for Abstracts checklist | 3 |
| **INTRODUCTION** |  |  |  |
| **Rationale** | 3 | Describe the rationale for the review in the context of existing knowledge. | 5 |
| **Objectives** | 4 | Provide an explicit statement of the objective(s) or question(s) the review addresses. | 6 |
| **METHODS** |  |  |  |
| **Eligibility criteria** | 5 | Specify the inclusion and exclusion criteria for the review and how studies were grouped for the syntheses. | 7 |
| **Information sources** | 6 | Specify all databases, registers, websites, organizations, reference lists and other sources searched or consulted to identify studies. Specify the date when each source was last searched or consulted. | 6 |
| **Search strategy** | 7 | Present the full search strategies for all databases, registers, and websites, including any filters and limits used. | 6 |
| **Selection process** | 8 | Specify the methods used to decide whether a study met the inclusion criteria of the review, including how many reviewers screened each record and each report retrieved, whether they worked independently, and if applicable, details of automation tools used in the process. | 7 |
| **Data collection process** | 9 | Specify the methods used to collect data from reports, including how many reviewers collected data from each report, whether they worked independently, any processes for obtaining or confirming data from study investigators, and if applicable, details of automation tools used in the process. | 7 |
| **Data items** | 10a | List and define all outcomes for which data were sought. Specify whether all results that were compatible with each outcome domain in each study were sought (e.g., for all measures, time points, analyses), and if not, the methods used to decide which results to collect. | 8 |
|  | 10b | List and define all other variables for which data were sought (e.g., participant and intervention characteristics, funding sources). Describe any assumptions made about any missing or unclear information. | 8 |
| **Study risk of bias assessment** | 11 | Specify the methods used to assess risk of bias in the included studies, including details of the tool(s) used, how many reviewers assessed each study and whether they worked independently, and if applicable, details of automation tools used in the process. | 8 |
| **Effect measures** | 12 | Specify for each outcome the effect measure(s) (e.g., risk ratio, mean difference) used in the synthesis or presentation of results. | 8 |
| **Synthesis methods** | 13a | Describe the processes used to decide which studies were eligible for each synthesis (e.g., tabulating the study intervention characteristics and comparing against the planned groups for each synthesis (item 5)). | 9 |
|  | 13b | Describe any methods required to prepare the data for presentation or synthesis, such as handling of missing summary statistics, or data conversions. | 8-9 |
|  | 13c | Describe any methods used to tabulate or visually display results of individual studies and syntheses. | 8-9 |
|  | 13d | Describe any methods used to synthesize results and provide a rationale for the choice(s). If meta-analysis was performed, describe the model(s), method(s) to identify the presence and extent of statistical heterogeneity, and software package(s) used. | 8-9 |
|  | 13e | Describe any methods used to explore possible causes of heterogeneity among study results (e.g., subgroup analysis, meta-regression). | 9 |
|  | 13f | Describe any sensitivity analyses conducted to assess robustness of the synthesized results. | 9 |
| **Reporting bias assessment** | 14 | Describe any methods used to assess risk of bias due to missing results in a synthesis (arising from reporting biases). | 8 |
| **Certainty assessment** | 15 | Describe any methods used to assess certainty (or confidence) in the body of evidence for an outcome. | 8 |
| **RESULTS** |  |  |  |
| **Study selection** | 16a | Describe the results of the search and selection process, from the number of records identified in the search to the number of studies included in the review, ideally using a flow diagram. | 9 |
|  | 16b | Cite studies that might appear to meet the inclusion criteria, but which were excluded, and explain why they were excluded. | - |
| **Study characteristics** | 17 | Cite each included study and present its characteristics. | 9 |
| **Risk of bias in studies** | 18 | Present assessments of risk of bias for each included study. | 12 |
| **Results of individual studies** | 19 | For all outcomes, present, for each study: (a) summary statistics for each group (where appropriate) and (b) an effect estimates and its precision (e.g., confidence/credible interval), ideally using structured tables or plots. | 9-11 |
| **Results of syntheses** | 20a | For each synthesis, briefly summarize the characteristics and risk of bias among contributing studies. | 10 |
|  | 20b | Present results of all statistical syntheses conducted. If meta-analysis was done, present for each the summary estimate and its precision (e.g., confidence/credible interval) and measures of statistical heterogeneity. If comparing groups, describe the direction of the effect. | 9-11 |
|  | 20c | Present results of all investigations of possible causes of heterogeneity among study results. | 9-11 |
|  | 20d | Present results of all sensitivity analyses conducted to assess the robustness of the synthesized results. | 10-11 |
| **Reporting biases** | 21 | Present assessments of risk of bias due to missing results (arising from reporting biases) for each synthesis assessed. | 12 |
| **Certainty of evidence** | 22 | Present assessments of certainty (or confidence) in the body of evidence for each outcome assessed. | 12 |
| **DISCUSSION** |  |  |  |
| **Discussion** | 23a | Provide a general interpretation of the results in the context of other evidence. | 12-15 |
|  | 23b | Discuss any limitations of the evidence included in the review. | 15 |
|  | 23c | Discuss any limitations of the review processes used. | - |
|  | 23d | Discuss implications of the results for practice, policy, and future research. | 15 |
| **OTHER INFORMATION** |  |  |  |
| **Registration and protocol** | 24a | Provide registration information for the review, including register name and registration number, or state that the review was not registered. | 6 |
|  | 24b | Indicate where the review protocol can be accessed, or state that a protocol was not prepared. | - |
|  | 24c | Describe and explain any amendments to information provided at registration or in the protocol. | No amendments |
| **Support** | 25 | Describe sources of financial or non-financial support for the review, and the role of the funders or sponsors in the review. | No Financial support |
| **Competing interests** | 26 | Declare any competing interests of review authors. | No competing interest |
| **Availability of data, code and other materials** | 27 | Report which of the following are publicly available and where they can be found template data collection forms; data extracted from included studies; data used for all analyses; analytic code; any other materials used in the review. | Data used for all analysis |

^Abbreviations: MS, manuscript; sup., supplement.^

**Table S2:** *PRISMA Abstract Checklist*

| **Topic** | **No.** | **Item** | **Reported?** |
| --- | --- | --- | --- |
| **TITLE** |  |  |  |
| **Title** | 1 | Identify the report as a systematic review. | Yes |
| **BACKGROUND** |  |  |  |
| **Objectives** | 2 | Provide an explicit statement of the main objective(s) or question(s) the review addresses. | Yes |
| **METHODS** |  |  |  |
| **Eligibility criteria** | 3 | Specify the inclusion and exclusion criteria for the review. | Yes |
| **Information sources** | 4 | Specify the information sources (e.g., databases, registers) used to identify studies and the date when each was last searched. | Yes |
| **Risk of bias** | 5 | Specify the methods used to assess risk of bias in the included studies. | Yes |
| **Synthesis of results** | 6 | Specify the methods used to present and synthesize results. | Yes |
| **RESULTS** |  |  |  |
| **Included studies** | 7 | Give the total number of included studies and participants and summarize relevant characteristics of studies. | Yes |
| **Synthesis of results** | 8 | Present results for main outcomes, preferably indicating the number of included studies and participants for each. If meta-analysis was done, report the summary estimate and confidence/credible interval. If comparing groups, indicate the direction of the effect (i.e., which group is favored). | Yes |
| **DISCUSSION** |  |  |  |
| **Limitations of evidence** | 9 | Provide a brief summary of the limitations of the evidence included in the review (e.g., study risk of bias, inconsistency and imprecision). | Yes |
| **Interpretation** | 10 | Provide a general interpretation of the results and important implications. | Yes |
| **OTHER** |  |  |  |
| **Funding** | 11 | Specify the primary source of funding for the review. | No |
| **Registration** | 12 | Provide the register name and registration number. | Yes |

**Table S3:** *Search Strategy*

| **Database** | **Filters applied** | **Results** | **Search strategy** |
| --- | --- | --- | --- |
| Pubmed | None | 33 | ("Enlicitide"[All Fields] OR ("mk 0616"[Supplementary Concept] OR "mk 0616"[All Fields] OR "mk 0616"[All Fields])) AND ("hypercholesterolaemia"[All Fields] OR "hypercholesterolemia"[MeSH Terms] OR "hypercholesterolemia"[All Fields] OR "hypercholesterolaemias"[All Fields] OR "hypercholesterolemias"[All Fields]) AND "LDL-C"[All Fields] AND ("pcsk9 inhibitors"[Pharmacological Action] OR "pcsk9 inhibitors"[Supplementary Concept] OR "pcsk9 inhibitors"[All Fields] OR "pcsk9 inhibitors"[MeSH Terms] OR ("pcsk9"[All Fields] AND "inhibitors"[All Fields])) AND ("heart disease risk factors"[MeSH Terms] OR ("heart"[All Fields] AND "disease"[All Fields] AND "risk"[All Fields] AND "factors"[All Fields]) OR "heart disease risk factors"[All Fields] OR ("cardiovascular"[All Fields] AND "risk"[All Fields]) OR "cardiovascular risk"[All Fields]) |
| Embase | None | 98 | (('Enlicitide':ti,ab,kw OR 'mk 0616':ti,ab,kw OR 'MK-0616':ti,ab,kw) AND ('hypercholesterolaemia':ti,ab,kw OR 'hypercholesterolemia'/exp OR 'hypercholesterolemia':ti,ab,kw OR 'hypercholesterolaemias':ti,ab,kw OR 'hypercholesterolemias':ti,ab,kw OR 'Hypercholesterolemia':ti,ab,kw) AND ('LDL-C':ti,ab,kw) AND  ('pcsk9 inhibitors'/exp OR 'pcsk9 inhibitors':ti,ab,kw OR 'PCSK9 inhibitors':ti,ab,kw OR (pcsk9:ti,ab,kw AND inhibitors:ti,ab,kw))  AND ('heart disease risk factors'/exp OR (heart:ti,ab,kw AND disease:ti,ab,kw AND risk:ti,ab,kw AND factors:ti,ab,kw) OR 'heart disease risk factors':ti,ab,kw OR (cardiovascular:ti,ab,kw AND risk:ti,ab,kw) OR 'cardiovascular risk':ti,ab,kw OR 'Cardiovascular risk':ti,ab,kw)) OR (('Enlicitide':ti,ab,kw OR 'MK-0616':ti,ab,kw) AND ('Hypercholesterolemia':ti,ab,kw) AND  ('LDL-C':ti,ab,kw) AND ('PCSK9 inhibitors':ti,ab,kw) AND ('Cardiovascular risk':ti,ab,kw)) OR (('Enlicitide':ti,ab,kw OR 'MK-0616':ti,ab,kw) AND ('Hypercholesterolemia':ti,ab,kw)) |
| Scopus | None | 114 | ALL ((enlicitide OR "MK-0616" OR "MK 0616") AND (hypercholesterolemia OR hypercholesterolaemia) AND ("LDL-C" OR "low-density lipoprotein cholesterol") AND ("PCSK9 inhibitor*" OR "proprotein convertase subtilisin/kexin type 9 inhibitor*") AND ("cardiovascular risk" OR "cardiovascular disease risk factor*" OR "heart disease risk factor*")) |
| Google scholar | None | 184 | “Enlicitide” OR “MK-0616” AND “Hypercholesterolemia” AND “LDL-C” AND “PCSK9 inhibitors” AND “Cardiovascular risk” |
| Clinical Trial.gov | None | 29 | “Enlicitide” OR “MK-0616” AND “Hypercholesterolemia” |

**Table S4:** *Grade Assessment*

| **Certainty assessment** | | | | | | | **№ of patients** | | **Effect** | | **Certainty** | **Importance** |
| --- | --- | --- | --- | --- | --- | --- | --- | --- | --- | --- | --- | --- |
| **№ of studies** | **Study design** | **Risk of bias** | **Inconsistency** | **Indirectness** | **Imprecision** | **Other considerations** | **Enlicitide** | **Placebo** | **Relative (95% CI)** | **Absolute (95% CI)** |  |  |
| **Percentage change from baseline in LDL-C levels** | | | | | | | | | | | | |
| **3** | **randomised trials** | **not serious** | **not serious** | **serious** | **not serious** | **none** | **2269** | **1068** | **-** | **MD 56.19 lower (64.14 lower to 48.25 lower)** | **⨁⨁⨁◯ Moderate** |  |
| **Percentage change from baseline in Non-HDL-C** | | | | | | | | | | | | |
| **3** | **randomised trials** | **not serious** | **not serious** | **serious** | **not serious** | **none** | **2270** | **1068** | **-** | **MD 51.36 lower (58.83 lower to 43.9 lower)** | **⨁⨁⨁◯ Moderate** |  |
| **Percentage change from baseline in Apolipoprotein-B** | | | | | | | | | | | | |
| **3** | **randomised trials** | **not serious** | **not serious** | **serious** | **not serious** | **none** | **2266** | **1059** | **-** | **MD 47.09 lower (53.51 lower to 40.66 lower)** | **⨁⨁⨁◯ Moderate** |  |
| **Percentage change from baseline in Lipoprotein(a)** | | | | | | | | | | | | |
| **3** | **randomised trials** | **not serious** | **not serious** | **serious** | **not serious** | **none** | **2261** | **1056** | **-** | **MD 20.1 lower (25.46 lower to 14.75 lower)** | **⨁⨁⨁◯ Moderate** |  |
| **Percentage change from baseline in Triglycerides** | | | | | | | | | | | | |
| **2** | **randomised trials** | **not serious** | **not serious** | **serious** | **not serious** | **none** | **2078** | **974** | **-** | **13.12 lower (17.65 lower to 8.58 lower)** | **⨁⨁⨁◯ Moderate** |  |
| **Any Adverse event** | | | | | | | | | | | | |
| **3** | **randomised trials** | **serious^a^** | **not serious** | **serious** | **not serious** | **none** | **1401/2137 (65.6%)** | **679/1070 (63.5%)** | **RR 1.03 (1.02 to 1.04)** | **19 more per 1,000 (from 13 more to 25 more)** | **⨁⨁◯◯ Low^a^** |  |
| **Serious Adverse Event** | | | | | | | | | | | | |
| **3** | **randomised trials** | **serious** | **not serious** | **serious** | **very serious** | **none** | **200/2137 (9.4%)** | **120/1070 (11.2%)** | **RR 0.84 (0.71 to 1.00)** | **18 fewer per 1,000 (from 33 fewer to 0 fewer)** | **⨁◯◯◯ Very low** |  |
| **Moderate to severe Adverse event** | | | | | | | | | | | | |
| **3** | **randomised trials** | **serious** | **not serious** | **serious** | **serious** | **none** | **711/2137 (33.3%)** | **347/1070 (32.4%)** | **RR 1.02 (0.97 to 1.08)** | **6 more per 1,000 (from 10 fewer to 26 more)** | **⨁◯◯◯ Very low** |  |
| **Discontinuation due to adverse events** | | | | | | | | | | | | |
| **3** | **randomised trials** | **serious** | **not serious** | **serious** | **serious** | **none** | **64/2137 (3.0%)** | **43/1070 (4.0%)** | **RR 0.77 (0.57 to 1.05)** | **9 fewer per 1,000 (from 17 fewer to 2 more)** | **⨁◯◯◯ Very low** |  |
| **All Cause mortality** | | | | | | | | | | | | |
| **3** | **randomised trials** | **serious** | **not serious** | **serious** | **very serious** | **none** | **14/2137 (0.7%)** | **7/1070 (0.7%)** | **RR 1.01 (0.44 to 2.33)** | **0 fewer per 1,000 (from 4 fewer to 9 more)** | **⨁◯◯◯ Very low** |  |

**CI: confidence interval; MD: mean difference; RR: risk ratio**

**Explanations**

**a. one of the 2 studies in this pooled outcome have some concerns when risk of biasness was assessed, due to which the overall grading is marked as serious and grading is downgraded by 1**

**Figure S1:** *Forest plot of subgroup analysis stratified by dose for percentage change from baseline in LDL-C.*

**Figure S2:** *Forest plot of subgroup analysis stratified by treatment durations for percentage change from baseline in LDL-C.*

**Figure S3:** *Sensitivity analysis for percentage change from baseline in LDL-C.*

**Figure S4:** *Leave-one-out analysis* *for percentage change from baseline in LDL-C.*

**Figure S5:** *Forest plot of subgroup analysis stratified by dose for percentage change from baseline in Non-HDL-C.*

**Figure S6:** *Forest plot of subgroup analysis stratified by treatment durations for percentage change from baseline in Non-HDL-C.*

**Figure S7:** *Sensitivity analysis for percentage change from baseline in Non-LDL-C.*

**Figure S8:** *Leave-one-out analysis for percentage change from baseline in Non-LDL-C.*

**Figure S9:** *Forest plot of subgroup analysis stratified by dose for percentage change from baseline in ApoB.*

**Figure S10:** *Forest plot of subgroup analysis stratified by treatment durations for percentage change from baseline in ApoB.*

**Figure S11:** *Sensitivity analysis for percentage change from baseline in ApoB.*

**Figure S12:** *Leave-one-out analysis for percentage change from baseline in ApoB.*

**Figure S13:** *Forest plot of subgroup analysis stratified by dose for percentage change from baseline in Lipoprotein(a).*

**Figure S14:** *Forest plot of subgroup analysis stratified by treatment durations for percentage change from baseline in Lipoprotein(a).*

**Figure S15:** *Sensitivity analysis for percentage change from baseline in Lipoprotein(a).*

**Figure S16:** *Forest plot of subgroup analysis stratified by treatment durations for percentage change from baseline in Triglycerides.*

**Figure S17:** *Risk of Bias Summary Plot*
